# {μ-6,6′-Dimeth­oxy-2,2′-[propane-1,3-diylbis(nitrilo­methyl­idyne)]diphenolato}trinitratocopper(II)europium(III)

**DOI:** 10.1107/S1600536808026329

**Published:** 2008-08-30

**Authors:** Jing-Chun Xing, Jing-Hua Wang, Peng-Fei Yan, Guang-Ming Li

**Affiliations:** a School of Chemistry and Materials Science, Heilongjiang University, Harbin 150080, People’s Republic of China; b Department of Anesthesiology of the Second Affiliated Hospital, Harbin Medical University, Harbin 150086, People’s Republic of China

## Abstract

In the title complex, [CuEu(C_19_H_20_N_2_O_4_)(NO_3_)_3_], the Cu^II^ ion is four-coordinated in a square-planar geometry by two O atoms and two N atoms of the deprotonated Schiff base. The Eu^III^ atom is ten-coordinate, chelated by three nitrate groups and linked to the four O atoms of the deprotonated Schiff base.

## Related literature

For copper–lanthanide complexes of the same Schiff base, see: Elmali & Elerman (2003 ▶); Elmali & Elerman (2004 ▶).
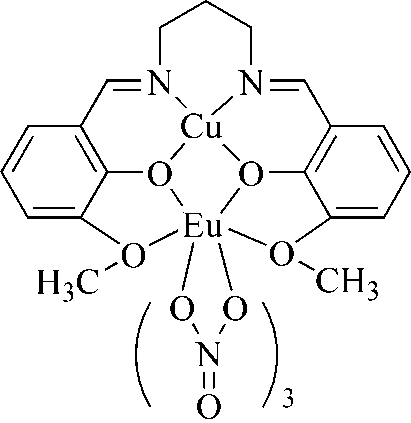


## Experimental

### 

#### Crystal data

[CuEu(C_19_H_20_N_2_O_4_)(NO_3_)_3_]
*M* *_r_* = 741.90
Monoclinic, 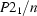

*a* = 11.638 (2) Å
*b* = 14.680 (3) Å
*c* = 14.853 (3) Å
β = 101.52 (3)°
*V* = 2486.5 (9) Å^3^
*Z* = 4
Mo *K*α radiation
μ = 3.43 mm^−1^
*T* = 291 (2) K
0.21 × 0.20 × 0.19 mm

#### Data collection

Rigaku R-AXIS RAPID diffractometer
Absorption correction: multi-scan (*ABSCOR*; Higashi, 1995 ▶) *T*_min_ = 0.527, *T*_max_ = 0.568 (expected range = 0.484–0.521)
23524 measured reflections
5660 independent reflections
5072 reflections with *I* > 2σ(*I*)
*R*_int_ = 0.031

#### Refinement

*R*[*F* ^2^ > 2σ(*F* ^2^)] = 0.024
*wR*(*F* ^2^) = 0.058
*S* = 1.06
5660 reflections
354 parameters
6 restraints
H-atom parameters constrained
Δρ_max_ = 0.72 e Å^−3^
Δρ_min_ = −0.45 e Å^−3^

### 

Data collection: *RAPID-AUTO* (Rigaku, 1998 ▶); cell refinement: *RAPID-AUTO*; data reduction: *CrystalStructure* (Rigaku/MSC, 2002 ▶); program(s) used to solve structure: *SHELXS97* (Sheldrick, 2008 ▶); program(s) used to refine structure: *SHELXL97* (Sheldrick, 2008 ▶); molecular graphics: *SHELXTL* (Sheldrick, 2008 ▶); software used to prepare material for publication: *SHELXL97*.

## Supplementary Material

Crystal structure: contains datablocks I, global. DOI: 10.1107/S1600536808026329/fj2142sup1.cif

Structure factors: contains datablocks I. DOI: 10.1107/S1600536808026329/fj2142Isup2.hkl

Additional supplementary materials:  crystallographic information; 3D view; checkCIF report

## Figures and Tables

**Fig. 1.**
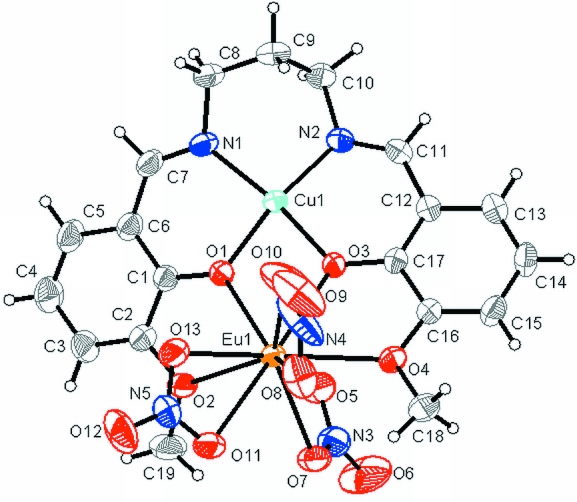
The molecular structure of (I), showing 40% probability displacement ellipsoids.

## supplementary crystallographic information

### Comment

As shown in Fig. 1, the hexadentate Schiff base ligand links Cu and Eu atoms into a dinuclear complex through two phenolate O atoms, which is similar with the bonding reported for another copper-lanthanide complex of the same ligand (Elmali & Elerman, 2003, 2004). The Eu^III^ centre in (I) is ten-coordinated by four oxygen atoms from the ligand and six oxygen atoms from three nitrate ions. The Cu^II^ center is four-coordinate by two nitrogen atoms and two oxygen atoms from the ligand.

### Experimental

The title complex was obtained by the treatment of copper(II) acetate monohydrate (0.0499 g, 0.25 mmol) with the Schiff base (0.0855 g, 0.25 mmol) in methanol (25 ml)at room temperature. Then the mixture was refluxed for 3 h after the addition of europium (III) nitrate hexahydrate (0.1117 g, 0.25 mmol). The reaction mixture was cooled and filtered; diethyl ether was allowed to diffuse slowly into the solution of the filtrate. Single crystals were obtained after several days. Analysis calculated for C_19_H_20_CuN_5_O_13_Eu: C, 30.78; H, 2.76; Cu, 8.50; N, 9.38; Eu, 20.58; found: C, 30.73; H, 2.70; Cu, 8.56; N, 9.44; Eu, 20.61%.

### Refinement

H atoms bound to C atoms were placed in calculated positions and treated as riding on their parent atoms, with C—H = 0.93 Å (aromatic C), C—H = 0.97 Å (methylene C), and with *U*_iso_(H) = 1.2Ueq(C) or C—H = 0.96 Å (methly C) and with *U*_iso_(H) = 1.5Ueq(C).

### Crystal data

**Table:**

[CuEu(C_19_H_20_N_2_O_4_)(NO_3_)_3_]	*F*_000_ = 1460
*M**_r_* = 741.90	*D*_x_ = 1.982 Mg m^−^^3^
Monoclinic, *P*2_1_/*n*	Mo *K*α radiation λ = 0.71073 Å
Hall symbol: -P 2yn	Cell parameters from 17062 reflections
*a* = 11.638 (2) Å	θ = 3.0–27.5º
*b* = 14.680 (3) Å	µ = 3.43 mm^−^^1^
*c* = 14.853 (3) Å	*T* = 291 (2) K
β = 101.52 (3)º	Block, red
*V* = 2486.5 (9) Å^3^	0.21 × 0.20 × 0.19 mm
*Z* = 4	

### Data collection

**Table:**

Rigaku R-AXIS RAPID diffractometer	5660 independent reflections
Radiation source: fine-focus sealed tube	5072 reflections with *I* > 2σ(*I*)
Monochromator: graphite	*R*_int_ = 0.031
Detector resolution: 10.000 pixels mm^-1^	θ_max_ = 27.5º
*T* = 291(2) K	θ_min_ = 3.1º
ω scans	*h* = −15→13
Absorption correction: multi-scan(ABSCOR; Higashi, 1995)	*k* = −19→19
*T*_min_ = 0.527, *T*_max_ = 0.568	*l* = −19→19
23524 measured reflections	

### Refinement

**Table:**

Refinement on *F*^2^	Secondary atom site location: difference Fourier map
Least-squares matrix: full	Hydrogen site location: inferred from neighbouring sites
*R*[*F*^2^ > 2σ(*F*^2^)] = 0.024	H-atom parameters constrained
*wR*(*F*^2^) = 0.058	*w* = 1/[σ^2^(*F*_o_^2^) + (0.0213*P*)^2^ + 2.281*P*] where *P* = (*F*_o_^2^ + 2*F*_c_^2^)/3
*S* = 1.06	(Δ/σ)_max_ = 0.002
5660 reflections	Δρ_max_ = 0.72 e Å^−^^3^
354 parameters	Δρ_min_ = −0.44 e Å^−^^3^
6 restraints	Extinction correction: none
Primary atom site location: structure-invariant direct methods	

### Special details

**Table:**

Geometry. All e.s.d.'s (except the e.s.d. in the dihedral angle between two l.s. planes) are estimated using the full covariance matrix. The cell e.s.d.'s are taken into account individually in the estimation of e.s.d.'s in distances, angles and torsion angles; correlations between e.s.d.'s in cell parameters are only used when they are defined by crystal symmetry. An approximate (isotropic) treatment of cell e.s.d.'s is used for estimating e.s.d.'s involving l.s. planes.
Refinement. Refinement of *F*^2^ against ALL reflections. The weighted *R*-factor *wR* and goodness of fit *S* are based on *F*^2^, conventional *R*-factors *R* are based on *F*, with *F* set to zero for negative *F*^2^. The threshold expression of *F*^2^ > σ(*F*^2^) is used only for calculating *R*-factors(gt) *etc*. and is not relevant to the choice of reflections for refinement. *R*-factors based on *F*^2^ are statistically about twice as large as those based on *F*, and *R*- factors based on ALL data will be even larger.

### Fractional atomic coordinates and isotropic or equivalent isotropic displacement parameters (Å^2^)

**Table:**

	*x*	*y*	*z*	*U*_iso_*/*U*_eq_	
C1	0.9061 (2)	0.36789 (18)	−0.06244 (19)	0.0317 (6)	
C2	0.7932 (3)	0.3919 (2)	−0.10789 (19)	0.0345 (6)	
C3	0.7755 (3)	0.4599 (2)	−0.1732 (2)	0.0449 (7)	
H1	0.6999	0.4743	−0.2038	0.054*	
C4	0.8712 (3)	0.5068 (2)	−0.1931 (2)	0.0523 (9)	
H2	0.8594	0.5536	−0.2361	0.063*	
C5	0.9823 (3)	0.4844 (2)	−0.1499 (2)	0.0463 (8)	
H3	1.0456	0.5162	−0.1639	0.056*	
C6	1.0024 (3)	0.4136 (2)	−0.0841 (2)	0.0365 (6)	
C7	1.1218 (3)	0.3936 (2)	−0.0403 (2)	0.0397 (7)	
H4	1.1783	0.4344	−0.0518	0.048*	
C8	1.2885 (3)	0.3275 (2)	0.0496 (3)	0.0536 (9)	
H5	1.3080	0.3813	0.0876	0.064*	
H6	1.3290	0.3320	−0.0013	0.064*	
C9	1.3326 (3)	0.2449 (2)	0.1056 (3)	0.0511 (9)	
H7	1.3038	0.2462	0.1625	0.061*	
H8	1.4176	0.2469	0.1211	0.061*	
C10	1.2950 (3)	0.1573 (2)	0.0561 (3)	0.0488 (8)	
H9	1.2975	0.1638	−0.0085	0.059*	
H10	1.3483	0.1088	0.0815	0.059*	
C11	1.1568 (2)	0.05275 (19)	0.09083 (19)	0.0336 (6)	
H11	1.2194	0.0126	0.0954	0.040*	
C12	1.0502 (2)	0.01617 (18)	0.11234 (18)	0.0304 (6)	
C13	1.0533 (3)	−0.0754 (2)	0.1430 (2)	0.0380 (7)	
H12	1.1202	−0.1105	0.1442	0.046*	
C14	0.9582 (3)	−0.1123 (2)	0.1709 (2)	0.0458 (8)	
H13	0.9602	−0.1727	0.1899	0.055*	
C15	0.8589 (3)	−0.0602 (2)	0.1710 (2)	0.0413 (7)	
H14	0.7953	−0.0853	0.1914	0.050*	
C16	0.8541 (2)	0.02870 (19)	0.14095 (19)	0.0321 (6)	
C17	0.9492 (2)	0.06820 (17)	0.10940 (17)	0.0282 (5)	
C18	0.6700 (3)	0.0552 (3)	0.1857 (3)	0.0560 (9)	
H15	0.6339	0.0016	0.1553	0.084*	
H16	0.6119	0.1019	0.1839	0.084*	
H17	0.7039	0.0408	0.2484	0.084*	
C19	0.5899 (3)	0.3491 (3)	−0.1377 (3)	0.0647 (11)	
H18	0.5650	0.4115	−0.1394	0.097*	
H19	0.5363	0.3122	−0.1121	0.097*	
H20	0.5913	0.3287	−0.1988	0.097*	
Cu1	1.05535 (3)	0.22819 (2)	0.03974 (2)	0.03036 (8)	
Eu1	0.762074 (11)	0.247433 (8)	0.068399 (9)	0.02785 (5)	
N1	1.1594 (2)	0.32671 (17)	0.01249 (18)	0.0382 (6)	
N2	1.1746 (2)	0.13432 (16)	0.06616 (17)	0.0341 (5)	
N3	0.6075 (3)	0.12249 (19)	−0.0485 (2)	0.0504 (7)	
N4	0.8226 (5)	0.2655 (3)	0.2660 (2)	0.0872 (15)	
N5	0.6480 (2)	0.42062 (17)	0.09471 (18)	0.0433 (6)	
O1	0.91672 (16)	0.30177 (13)	0.00059 (14)	0.0353 (4)	
O2	0.70508 (18)	0.34136 (15)	−0.08175 (14)	0.0399 (5)	
O3	0.93757 (16)	0.15332 (12)	0.07838 (13)	0.0324 (4)	
O4	0.76063 (18)	0.08689 (14)	0.13955 (15)	0.0390 (5)	
O5	0.7096 (2)	0.13933 (16)	−0.06160 (16)	0.0478 (5)	
O6	0.5492 (3)	0.0630 (2)	−0.0900 (3)	0.1037 (13)	
O7	0.57208 (18)	0.17165 (15)	0.01090 (16)	0.0445 (5)	
O8	0.7188 (3)	0.25602 (16)	0.2249 (2)	0.0633 (8)	
O9	0.8490 (5)	0.2703 (3)	0.3497 (2)	0.1377 (17)	
O10	0.8992 (3)	0.2700 (2)	0.2174 (2)	0.0809 (10)	
O11	0.58719 (19)	0.35057 (16)	0.07275 (17)	0.0478 (5)	
O12	0.6061 (3)	0.49507 (18)	0.1043 (2)	0.0779 (9)	
O13	0.75861 (19)	0.41086 (15)	0.10619 (17)	0.0457 (5)	

### Atomic displacement parameters (Å^2^)

**Table:**

	*U*^11^	*U*^22^	*U*^33^	*U*^12^	*U*^13^	*U*^23^
C1	0.0326 (14)	0.0304 (13)	0.0328 (14)	0.0001 (11)	0.0084 (11)	0.0008 (11)
C2	0.0338 (15)	0.0357 (14)	0.0341 (14)	−0.0031 (12)	0.0074 (12)	0.0002 (12)
C3	0.0492 (19)	0.0455 (18)	0.0374 (16)	0.0018 (15)	0.0022 (14)	0.0098 (13)
C4	0.062 (2)	0.052 (2)	0.0429 (18)	−0.0013 (17)	0.0104 (16)	0.0185 (15)
C5	0.052 (2)	0.0428 (17)	0.0477 (18)	−0.0078 (15)	0.0186 (16)	0.0115 (14)
C6	0.0382 (16)	0.0335 (14)	0.0408 (16)	−0.0030 (12)	0.0149 (13)	0.0017 (12)
C7	0.0350 (16)	0.0353 (15)	0.0528 (18)	−0.0075 (13)	0.0186 (14)	0.0013 (13)
C8	0.0262 (16)	0.0448 (19)	0.089 (3)	−0.0044 (14)	0.0089 (17)	−0.0001 (18)
C9	0.0259 (16)	0.059 (2)	0.067 (2)	−0.0044 (14)	0.0062 (15)	0.0024 (17)
C10	0.0306 (16)	0.0420 (17)	0.079 (2)	0.0051 (14)	0.0225 (16)	0.0063 (16)
C11	0.0298 (14)	0.0363 (14)	0.0340 (14)	0.0067 (12)	0.0052 (11)	0.0010 (12)
C12	0.0329 (14)	0.0304 (13)	0.0280 (13)	0.0012 (11)	0.0065 (11)	−0.0008 (10)
C13	0.0431 (17)	0.0323 (14)	0.0382 (16)	0.0068 (13)	0.0070 (13)	0.0022 (12)
C14	0.057 (2)	0.0283 (14)	0.0526 (19)	−0.0006 (14)	0.0135 (16)	0.0089 (13)
C15	0.0448 (18)	0.0352 (15)	0.0463 (17)	−0.0081 (13)	0.0145 (14)	0.0057 (13)
C16	0.0310 (14)	0.0312 (14)	0.0343 (14)	−0.0015 (11)	0.0072 (11)	0.0011 (11)
C17	0.0323 (14)	0.0268 (12)	0.0254 (12)	−0.0026 (11)	0.0054 (10)	−0.0005 (10)
C18	0.0374 (18)	0.061 (2)	0.077 (3)	−0.0020 (16)	0.0289 (18)	0.0201 (19)
C19	0.0328 (18)	0.084 (3)	0.069 (2)	−0.0063 (18)	−0.0103 (17)	0.024 (2)
Cu1	0.02206 (16)	0.03009 (16)	0.03997 (18)	−0.00026 (13)	0.00866 (14)	0.00474 (14)
Eu1	0.02169 (8)	0.02888 (8)	0.03354 (8)	−0.00131 (5)	0.00688 (5)	−0.00045 (5)
N1	0.0247 (12)	0.0372 (13)	0.0539 (15)	−0.0038 (10)	0.0110 (11)	−0.0005 (11)
N2	0.0254 (12)	0.0361 (13)	0.0422 (13)	0.0030 (10)	0.0103 (10)	0.0021 (10)
N3	0.0429 (16)	0.0409 (15)	0.0632 (18)	−0.0032 (12)	0.0008 (14)	−0.0151 (13)
N4	0.119 (4)	0.100 (3)	0.0346 (16)	0.079 (3)	−0.003 (2)	−0.0102 (17)
N5	0.0479 (17)	0.0361 (14)	0.0454 (15)	0.0078 (12)	0.0084 (12)	0.0007 (11)
O1	0.0274 (10)	0.0341 (10)	0.0457 (11)	0.0018 (8)	0.0107 (9)	0.0132 (9)
O2	0.0270 (10)	0.0460 (12)	0.0437 (12)	−0.0020 (9)	0.0002 (9)	0.0098 (9)
O3	0.0269 (10)	0.0276 (9)	0.0447 (11)	0.0020 (8)	0.0122 (8)	0.0073 (8)
O4	0.0310 (11)	0.0382 (11)	0.0521 (12)	−0.0011 (9)	0.0181 (10)	0.0089 (9)
O5	0.0432 (13)	0.0474 (13)	0.0544 (13)	−0.0019 (11)	0.0136 (11)	−0.0141 (11)
O6	0.068 (2)	0.087 (2)	0.151 (3)	−0.0302 (18)	0.010 (2)	−0.070 (2)
O7	0.0293 (11)	0.0483 (13)	0.0551 (13)	−0.0027 (9)	0.0066 (10)	−0.0091 (11)
O8	0.088 (2)	0.0581 (16)	0.0499 (15)	0.0195 (14)	0.0294 (16)	0.0010 (12)
O9	0.166 (4)	0.192 (4)	0.0457 (17)	0.115 (3)	−0.003 (2)	−0.020 (2)
O10	0.0621 (19)	0.114 (3)	0.0561 (17)	0.0386 (18)	−0.0146 (15)	−0.0300 (17)
O11	0.0298 (11)	0.0466 (13)	0.0674 (15)	0.0017 (10)	0.0107 (10)	−0.0029 (11)
O12	0.089 (2)	0.0437 (14)	0.099 (2)	0.0295 (15)	0.0140 (18)	−0.0063 (15)
O13	0.0378 (12)	0.0380 (12)	0.0607 (14)	−0.0067 (9)	0.0087 (10)	−0.0057 (10)

### Geometric parameters (Å, °)

**Table:**

C1—O1	1.337 (3)	C16—O4	1.380 (3)
C1—C6	1.398 (4)	C16—C17	1.409 (4)
C1—C2	1.398 (4)	C17—O3	1.329 (3)
C2—C3	1.378 (4)	C18—O4	1.445 (3)
C2—O2	1.383 (3)	C18—H15	0.9600
C3—C4	1.392 (5)	C18—H16	0.9600
C3—H1	0.9300	C18—H17	0.9600
C4—C5	1.363 (5)	C19—O2	1.433 (4)
C4—H2	0.9300	C19—H18	0.9600
C5—C6	1.414 (4)	C19—H19	0.9600
C5—H3	0.9300	C19—H20	0.9600
C6—C7	1.441 (4)	Cu1—O3	1.9315 (18)
C7—N1	1.278 (4)	Cu1—O1	1.9320 (19)
C7—H4	0.9300	Cu1—N2	1.940 (2)
C8—N1	1.494 (4)	Cu1—N1	1.980 (2)
C8—C9	1.501 (5)	Eu1—O1	2.3694 (19)
C8—H5	0.9700	Eu1—O3	2.4457 (18)
C8—H6	0.9700	Eu1—O13	2.466 (2)
C9—C10	1.502 (5)	Eu1—O7	2.470 (2)
C9—H7	0.9700	Eu1—O8	2.478 (3)
C9—H8	0.9700	Eu1—O10	2.478 (3)
C10—N2	1.478 (4)	Eu1—O5	2.480 (2)
C10—H9	0.9700	Eu1—O11	2.548 (2)
C10—H10	0.9700	Eu1—O4	2.584 (2)
C11—N2	1.281 (4)	Eu1—O2	2.593 (2)
C11—C12	1.445 (4)	N3—O6	1.198 (4)
C11—H11	0.9300	N3—O5	1.266 (4)
C12—C17	1.395 (4)	N3—O7	1.271 (3)
C12—C13	1.418 (4)	N4—O9	1.222 (5)
C13—C14	1.368 (4)	N4—O8	1.249 (5)
C13—H12	0.9300	N4—O10	1.256 (6)
C14—C15	1.387 (5)	N5—O12	1.216 (3)
C14—H13	0.9300	N5—O11	1.254 (3)
C15—C16	1.376 (4)	N5—O13	1.273 (3)
C15—H14	0.9300		
			
O1—C1—C6	122.9 (3)	O1—Eu1—O3	61.31 (6)
O1—C1—C2	117.9 (2)	O1—Eu1—O13	79.49 (7)
C6—C1—C2	119.2 (3)	O3—Eu1—O13	125.96 (7)
C3—C2—O2	124.8 (3)	O1—Eu1—O7	135.09 (7)
C3—C2—C1	121.1 (3)	O3—Eu1—O7	116.46 (7)
O2—C2—C1	114.0 (2)	O13—Eu1—O7	117.56 (7)
C2—C3—C4	119.6 (3)	O1—Eu1—O8	134.09 (10)
C2—C3—H1	120.2	O3—Eu1—O8	107.21 (9)
C4—C3—H1	120.2	O13—Eu1—O8	73.82 (8)
C5—C4—C3	120.3 (3)	O7—Eu1—O8	90.65 (10)
C5—C4—H2	119.8	O1—Eu1—O10	85.77 (10)
C3—C4—H2	119.8	O3—Eu1—O10	68.91 (8)
C4—C5—C6	120.9 (3)	O13—Eu1—O10	72.77 (9)
C4—C5—H3	119.6	O7—Eu1—O10	137.83 (11)
C6—C5—H3	119.6	O8—Eu1—O10	51.04 (12)
C1—C6—C5	118.8 (3)	O1—Eu1—O5	88.53 (8)
C1—C6—C7	122.9 (3)	O3—Eu1—O5	76.02 (8)
C5—C6—C7	118.2 (3)	O13—Eu1—O5	141.92 (8)
N1—C7—C6	127.9 (3)	O7—Eu1—O5	51.56 (8)
N1—C7—H4	116.1	O8—Eu1—O5	134.29 (9)
C6—C7—H4	116.1	O10—Eu1—O5	142.67 (9)
N1—C8—C9	113.9 (3)	O1—Eu1—O11	119.29 (7)
N1—C8—H5	108.8	O3—Eu1—O11	174.77 (7)
C9—C8—H5	108.8	O13—Eu1—O11	50.70 (7)
N1—C8—H6	108.8	O7—Eu1—O11	67.17 (8)
C9—C8—H6	108.8	O8—Eu1—O11	68.45 (9)
H5—C8—H6	107.7	O10—Eu1—O11	105.86 (9)
C8—C9—C10	112.7 (3)	O5—Eu1—O11	109.04 (8)
C8—C9—H7	109.0	O1—Eu1—O4	123.26 (6)
C10—C9—H7	109.0	O3—Eu1—O4	62.20 (6)
C8—C9—H8	109.0	O13—Eu1—O4	142.41 (8)
C10—C9—H8	109.0	O7—Eu1—O4	69.64 (7)
H7—C9—H8	107.8	O8—Eu1—O4	69.12 (7)
N2—C10—C9	109.6 (3)	O10—Eu1—O4	79.18 (10)
N2—C10—H9	109.7	O5—Eu1—O4	73.38 (8)
C9—C10—H9	109.7	O11—Eu1—O4	117.46 (7)
N2—C10—H10	109.7	O1—Eu1—O2	62.67 (7)
C9—C10—H10	109.7	O3—Eu1—O2	114.81 (7)
H9—C10—H10	108.2	O13—Eu1—O2	70.45 (8)
N2—C11—C12	127.3 (3)	O7—Eu1—O2	83.38 (8)
N2—C11—H11	116.3	O8—Eu1—O2	135.61 (8)
C12—C11—H11	116.3	O10—Eu1—O2	134.86 (10)
C17—C12—C13	119.8 (3)	O5—Eu1—O2	71.93 (8)
C17—C12—C11	123.0 (2)	O11—Eu1—O2	68.74 (8)
C13—C12—C11	117.1 (3)	O4—Eu1—O2	144.61 (7)
C14—C13—C12	120.1 (3)	C7—N1—C8	114.6 (3)
C14—C13—H12	120.0	C7—N1—Cu1	122.6 (2)
C12—C13—H12	120.0	C8—N1—Cu1	122.8 (2)
C13—C14—C15	120.5 (3)	C11—N2—C10	117.0 (2)
C13—C14—H13	119.7	C11—N2—Cu1	124.85 (19)
C15—C14—H13	119.7	C10—N2—Cu1	118.15 (19)
C16—C15—C14	120.1 (3)	O6—N3—O5	121.1 (3)
C16—C15—H14	119.9	O6—N3—O7	122.8 (3)
C14—C15—H14	119.9	O5—N3—O7	116.1 (2)
C15—C16—O4	124.9 (3)	O9—N4—O8	121.7 (5)
C15—C16—C17	121.0 (3)	O9—N4—O10	121.3 (5)
O4—C16—C17	114.1 (2)	O8—N4—O10	117.0 (3)
O3—C17—C12	123.5 (2)	O12—N5—O11	123.3 (3)
O3—C17—C16	118.0 (2)	O12—N5—O13	120.2 (3)
C12—C17—C16	118.5 (2)	O11—N5—O13	116.5 (2)
O4—C18—H15	109.5	C1—O1—Cu1	124.84 (17)
O4—C18—H16	109.5	C1—O1—Eu1	124.78 (16)
H15—C18—H16	109.5	Cu1—O1—Eu1	110.09 (8)
O4—C18—H17	109.5	C2—O2—C19	117.1 (2)
H15—C18—H17	109.5	C2—O2—Eu1	116.77 (17)
H16—C18—H17	109.5	C19—O2—Eu1	126.1 (2)
O2—C19—H18	109.5	C17—O3—Cu1	127.52 (17)
O2—C19—H19	109.5	C17—O3—Eu1	125.29 (16)
H18—C19—H19	109.5	Cu1—O3—Eu1	107.10 (8)
O2—C19—H20	109.5	C16—O4—C18	116.2 (2)
H18—C19—H20	109.5	C16—O4—Eu1	120.07 (15)
H19—C19—H20	109.5	C18—O4—Eu1	123.57 (19)
O3—Cu1—O1	78.94 (8)	N3—O5—Eu1	95.64 (17)
O3—Cu1—N2	93.28 (9)	N3—O7—Eu1	95.94 (17)
O1—Cu1—N2	168.21 (10)	N4—O8—Eu1	96.1 (2)
O3—Cu1—N1	167.44 (9)	N4—O10—Eu1	95.9 (3)
O1—Cu1—N1	92.19 (9)	N5—O11—Eu1	94.71 (17)
N2—Cu1—N1	96.78 (10)	N5—O13—Eu1	98.11 (17)
